# Dichotomous Impact of Myc on rRNA Gene Activation and Silencing in B Cell Lymphomagenesis

**DOI:** 10.3390/cancers12103009

**Published:** 2020-10-16

**Authors:** Gaurav Joshi, Alexander Otto Eberhardt, Lisa Lange, René Winkler, Steve Hoffmann, Christian Kosan, Holger Bierhoff

**Affiliations:** 1 Center for Molecular Biomedicine (CMB), Institute of Biochemistry and Biophysics, Friedrich Schiller University Jena, Hans-Knöll-Str. 2, 07745 Jena, Germany; G.Joshi@imb-mainz.de (G.J.); alexander.eberhardt@uni-jena.de (A.O.E.); lisa.lange@uni-jena.de (L.L.); rene.winkler@uni-jena.de (R.W.); christian.kosan@uni-jena.de (C.K.); 2 Indian Institute of Science Education and Research (IISER), Dr. Homi Bhabha Road, Pune 411008, India; 3 Leibniz-Institute on Aging—Fritz Lipmann Institute (FLI), Beutenbergstrasse 11, 07745 Jena, Germany; steve.hoffmann@leibniz-fli.de

**Keywords:** cancer, lymphomagenesis, Myc, ribosomal RNA, gene transcription, epigenetic regulation, CpG methylation, genomic instability

## Abstract

**Simple Summary:**

B cell lymphomas mostly arise from malignant transformation of mature B cells and are typically driven by elevated levels of the oncoprotein Myc. Myc is a transcription factor regulating many protein-coding genes as well as the multicopy genes encoding ribosomal RNA (rRNA). The aim of this study was to understand, how Myc impacts rRNA genes in the course of B cell lymphomagenesis. Using a transgenic mouse model, we found that *Myc* and rRNA gene expression strongly increase upon tumor formation. Surprisingly, Myc also facilitates epigenetic silencing of a fraction of rRNA genes, thereby safeguarding genomic integrity in lymphoma cells. Together, the results show that Myc balances high activity and stability of rRNA genes. Perturbation of this equilibrium may be used as a therapeutic strategy.

**Abstract:**

A major transcriptional output of cells is ribosomal RNA (rRNA), synthesized by RNA polymerase I (Pol I) from multicopy rRNA genes (rDNA). Constitutive silencing of an rDNA fraction by promoter CpG methylation contributes to the stabilization of these otherwise highly active loci. In cancers driven by the oncoprotein Myc, excessive Myc directly stimulates rDNA transcription. However, it is not clear when during carcinogenesis this mechanism emerges, and how Myc-driven rDNA activation affects epigenetic silencing. Here, we have used the Eµ-*Myc* mouse model to investigate rDNA transcription and epigenetic regulation in Myc-driven B cell lymphomagenesis. We have developed a refined cytometric strategy to isolate B cells from the tumor initiation, promotion, and progression phases, and found a substantial increase of both *Myc* and rRNA gene expression only in established lymphoma. Surprisingly, promoter CpG methylation and the machinery for rDNA silencing were also strongly up-regulated in the tumor progression state. The data indicate a dichotomous role of oncogenic Myc in rDNA regulation, boosting transcription as well as reinforcing repression of silent repeats, which may provide a novel angle on perturbing Myc function in cancer cells.

## 1. Introduction

Cancer formation is a multistage process comprising the accumulation of molecular lesions that override cellular growth control. The complexity and heterogeneity of cancers make it difficult to discriminate driver and bystander events of malignant transformation. However, research over the last decades has uncovered several cellular capabilities that are typically acquired during carcinogenesis and are, therefore, coined “hallmarks of cancer” [1,2,3]. One of these hallmarks, i.e., sustained proliferative signaling, can be viewed as the most fundamental characteristic of cancer cells. A key factor integrating proliferation signals and transmitting them to target genes is the proto-oncoprotein Myc. Myc is a potent growth-promoting factor and the *Myc* gene is overexpressed in most cancers, either due to chromosomal translocation, amplification, or transcriptional dysregulation [4,5]. The first observation that *Myc* overexpression drives human carcinogenesis was made in Burkitt lymphoma, an aggressive B cell lymphoma, in which the *Myc* gene is translocated to the immunoglobulin loci [6,7].

Myc is a versatile transcription factor, which, together with its obligate binding partner Max, binds to thousands of gene promoters and enhancers, thus occupying about 10–15% of genomic loci [8,9,10]. Intriguingly, Myc/Max heterodimers do not only act on RNA polymerase II (Pol II) transcribed genes, but also stimulates the synthesis of housekeeping non-coding RNAs, e.g., rRNA and tRNA, by Pol I and Pol III, respectively [11,12,13,14]. Thereby, they promote the concerted action of all three RNA polymerases required for ribosome biogenesis, and in turn stimulate protein synthesis and cell growth [15]. This mechanism is a rate-limiting determinant of cancer initiation, but is also required for the subsequent promotion and progression phases of carcinogenesis [4,16]. However, when Myc’s intracellular levels and functions change during tumor development is so far not well understood. In contrast to Myc, Max is a stable and abundant protein, which is not deregulated in cancer, albeit it is essential for Myc-driven lymphomagenesis [17,18].

Most steps of ribosome biogenesis take place in nucleoli, the most prominent non-membrane bound organelles in the cell nucleus. An integral part of nucleoli are the rRNA gene arrays that are transcribed by Pol I into a precursor-rRNA (pre-rRNA) containing the 18S, 5.8S and 28S rRNA sequences. Transcription of rDNA accounts for the majority of cellular RNA synthesis and sets the pace for ribosome biogenesis. Thus, signaling and epigenetic pathways tightly control rDNA activity according to cell proliferation [19]. Moreover, a fraction of the repeats is constitutively repressed in somatic cells. This epigenetic silencing is mediated by the nucleolar remodeling complex (NoRC), comprising the proteins TIP5 and SNF2H as well as a long non-coding RNA termed pRNA. pRNA is a 150–250 nucleotides long Pol I transcript originating from a promoter upstream of the pre-rRNA start site and is essential for NoRC function [19,20]. NoRC binds together with pRNA to the rDNA promoter and serves as a scaffold for repressive chromatin modifiers, including DNA methyltransferases (DNMTs) [21]. CpG methylation of the rDNA promoter is incompatible with Pol I binding and, thus, abrogates transcription [22]. Several studies indicate that NoRC/pRNA-dependent silencing safeguards the integrity of rDNA and that loss of heterochromatin formation is linked to genome instability [19,20,23,24].

Cancer cells employ several mechanisms, including *Myc* overexpression, to enhance Pol I activity. A corresponding increase in nucleolar size has long been known as a hallmark of cancer [25,26,27]. However, elevated rDNA transcription bears the risk of increased DNA damage and derepression of silent repeats. In fact, rDNA rearrangements are prevalent in cancer [28], but it is not clear whether this is caused by transcriptional and epigenetic changes. In this study, we investigated Myc-dependent dysregulation of rRNA genes upon spontaneous B cell lymphomagenesis in the Eµ-*Myc* mouse model, which recapitulates many features of human Burkitt lymphoma [29,30]. Harboring a *Myc* transgene under the control of the Eµ enhancer, the model mimics the characteristic t(8:14) translocation and has been instrumental in dissecting the molecular pathology of lymphomagenesis [31,32]. We report that pre-malignant Eµ-*Myc* mice harbor two B cell populations that represent the initiation and promotion states of carcinogenesis. Whereas these states show only a moderate increase in both *Myc* and rRNA gene expression, strong up-regulation occurs upon tumor progression and maintenance. Counterintuitively, pre-malignancy and malignancy are both associated with enhanced rDNA promoter methylation. Given that the expression of DNMTs and pRNA parallels the gain in Myc levels during lymphomagenesis, we argue that Myc not only promotes rRNA synthesis, but also ensures NoRC-dependent rDNA silencing. Abrogation of silencing compromises rDNA integrity, thus constituting a so far unexplored vulnerability of Myc-driven cancers.

## 2. Results

### 2.1. Drastic Increase of Myc and rRNA Gene Expression upon Lymphoma Progression

**Figure 1:**
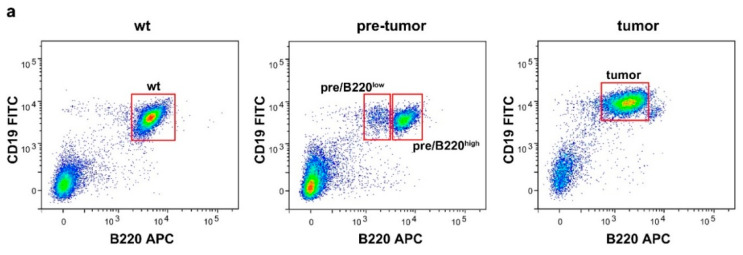

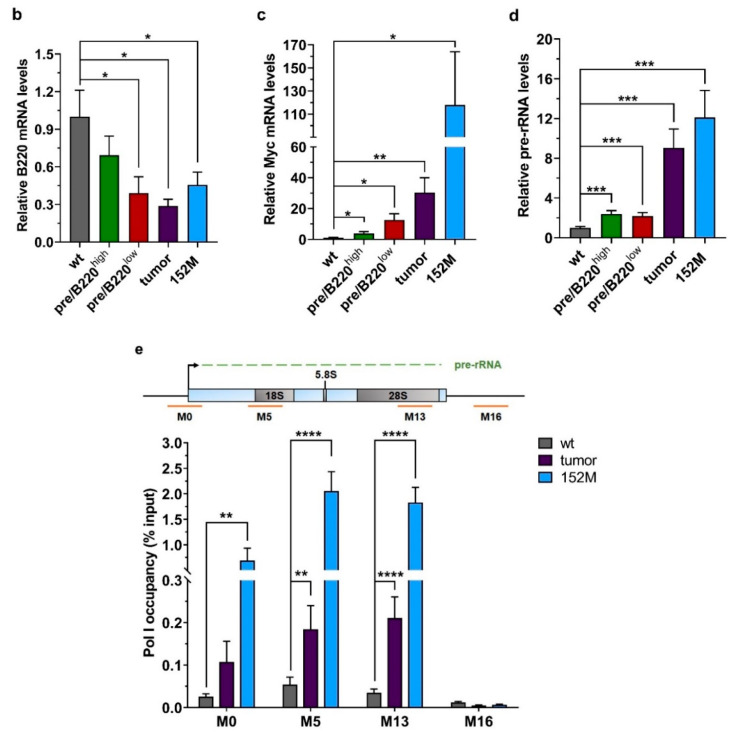
Up-regulation of Pol I transcription in Myc-driven lymphomagenesis. (**a**) Representative FACS plots of B cells isolated from wt mice (left) or pre-tumoral (middle) and tumoral (right) Eµ-*Myc* mice. Populations used for further analysis are boxed. (**b**–**d**) Expression of *B220*, *Myc* and rRNA genes in primary B cells from wt and Eµ-*Myc* mice as well as in the Eµ-*Myc* lymphoma cell line 152M. Transcript levels were measured after reverse transcription by qPCR and normalized to B2M mRNA levels. (**e**) Occupancy of the Pol I subunit RPA116 at rDNA in wt, tumor, and 152M cells. ChIP was followed by qPCR using rDNA amplicons indicated in the scheme above and enrichment was calculated relative to input. Statistical analysis was performed using two-way ANOVA with Tukey multiple comparison test. Data in (**b**–**e**) represent mean values ± SEM from at least three biological replicates. * *p* < 0.05, ** *p* < 0.01, *** *p* < 0.001, and **** *p* < 0.0001.

Development of B cell lymphoma in Eµ-*Myc* mice is preceded by a pre-malignant phase in which initiation and promotion of malignant transformation take place [29,30]. To compare the different stages of lymphomagenesis with healthy B cells, we isolated splenic B cells from 8–10 weeks old wild-type (wt) mice and from Eµ-*Myc* mice without any signs of tumor development. Additionally, tumor-bearing Eµ-*Myc* mice at the age of 10–12 weeks were sacrificed and B cells were isolated from infiltrated lymph nodes. Cytometric analysis with antibodies against B cell surface markers CD19 and B220 showed that wt B cells exhibit high levels of B220, whereas tumor B cells had reduced B220 levels (Figure 1a). Importantly, fluorescence-activated cell sorting (FACS) of cells from pre-malignant animals revealed the existence of two populations, one with high B220 surface levels akin to the wt state (pre/B220^high^), and one with low B220 expression resembling the tumor situation (pre/B220^low^) (Figure 1a). To validate the FACS results, we monitored expression of the *Ptprc* gene encoding B220 mRNA and found similarly high levels in wt and pre/B220^high^ cells (Figure 1b). In contrast, pre/B220^low^ and tumor B cells showed significantly weaker *Ptprc* expression, thus confirming the FACS results. In addition to the primary tumor cells, we also established an Eµ-*Myc* lymphoma cell line termed “152M” with B220 levels comparable to the primary tumor cells (Figure 1b). Previous studies reported progressively increasing *Myc* mRNA levels when comparing non-transgenic mice with pre-tumoral and tumoral Eµ-*Myc* mice [33,34]. Accordingly, our measurement of *Myc* expression confirmed this trend (Figure 1c). Moreover, we found gradual elevation of *Myc* mRNA levels in pre-tumoral animals from the initiation (pre/B220^high^) to promotion (pre/B220^low^) stage. Tumor B cells showed further augmented *Myc* expression, which was even exceeded in 152M cells, indicating that lymphoma maintenance is strongly dependent on Myc (Figure 1c). Given that Myc directly activates Pol I transcription [11,12], we next analyzed pre-rRNA synthesis in the course of lymphomagenesis. Compared to wt cells, pre-rRNA levels were elevated by ~2-fold in both pre-malignant states (Figure 1d). In contrast to this moderate effect, progression to the tumor stage was associated with a strong increase in rRNA synthesis rates, pre-rRNA being ~10-fold more abundant than in normal B cells. Similar to the *Myc* expression, pre-rRNA levels were highest in the in vitro cultured lymphoma cells (Figure 1d).

To confirm that pre-rRNA levels indeed reflect Pol I transcriptional activity, we analyzed rDNA occupancy of the second largest Pol I subunit RPA116 by chromatin immunoprecipitation (ChIP). Compared to wt B cells, primary and established tumor cells displayed markedly enhanced binding of Pol I to the promoter (amplicon M0) as well as along the gene body (M5 and M13) (Figure 1e). ChIP signals ceased in the intergenic spacer (M16), confirming the specificity of the anti-RPA116 antibody. Owing to the separation of pre-malignant B cells in pre/B220^high^ and pre/B220^low^ populations, we did not obtain sufficient cell material for ChIP analysis of these stages. However, the results in wt and tumor cells support the notion, that the excessively increased Myc levels induce a strong boost of rDNA transcription.

### 2.2. Hypertranscription of rDNA Coexists with Reinforced Epigenetic Silencing

**Figure 2:**
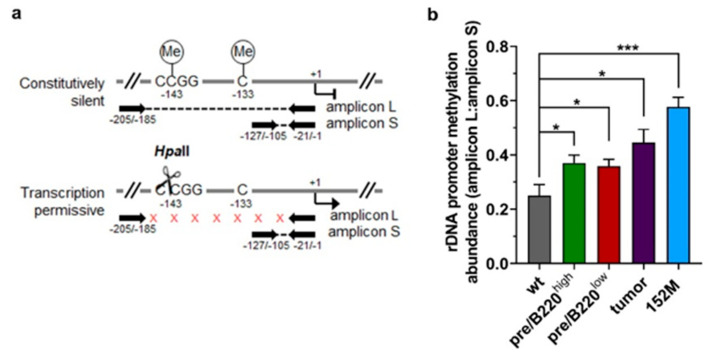

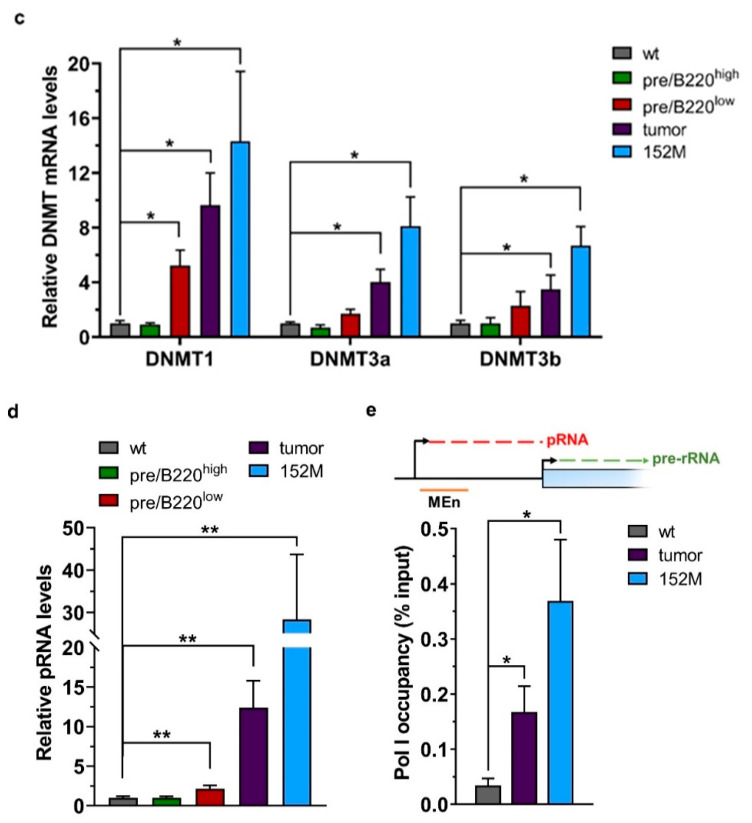
Epigenetic silencing of rDNA upon malignant transformation of B cells. (**a**) Scheme illustrating quantification of rDNA promoter methylation. After DNA digestion with *Hpa*II, qPCR for amplicons L and S provides the fraction of methylated promoters. (**b**) Ratio of promoter methylation in wt and Eµ-*Myc* B cells. (**c**,**d**) Levels of DNMT1, 3a and 3b mRNAs, and of pRNA, relative to B2M mRNA in the indicated B cell populations. (**e**) RPA116 occupancy at the rDNA enhancer region upstream of the pre-rRNA start site. Position of the rDNA region (MEn) amplified in qPCR is shown in the diagram. ChIP-enrichment was calculated relative to input and statistical significance was tested by one-way ANOVA with Tukey multiple comparisons. Bar graphs in (**b**–**e**) show mean values ± SEM from at least three biological replicates. * *p* < 0.05, ** *p* < 0.01, and *** *p* < 0.001.

Cancer cells can increase the transcriptional output of rDNA either by escalating the activity of permissive rRNA genes or by derepressing repeats formerly silenced by NoRC. Both strategies have been observed in prostate and cervical cancers, respectively [35,36]. To investigate whether enhanced pre-rRNA synthesis upon lymphomagenesis involves reactivation of silent rRNA genes, we monitored promoter methylation using the methylation-sensitive restriction enzyme *Hpa*II. The cytosine at position −143 of the mouse rDNA promoter is contained within an *Hpa*II target site and its methylation is indicative for the transcription-refractory state [37]. Restriction digestion was followed by qPCR with rDNA promoter-specific primer pairs either encompassing the *Hpa*II site (amplicon L) or not (amplicon S), and the relative CpG methylation frequency was determined by the amplicon L/S ratio (Figure 2a). Surprisingly, rDNA promoter methylation was more abundant in pre-tumoral and tumoral B cells than in wt B cells, the L/S ratio being ~1.5-fold higher in pre/B220^high^ and pre/B220^low^ cells, and even 1.7–2 times higher in lymphoma cells (Figure 2b). Given that *Hpa*II sensitivity is also caused by transcription-compatible 5-hydroxymethylation [38], we employed a method based on glucosylation and restriction digestion to interrogate 5-hydroxymethylcytosine levels at position -143. In all B cell states tested, this cytosine modification was barely detectable at rDNA promoters, indicating that it did not contribute to the observed methylation changes (Figure S1). Having shown that, despite rDNA hypertranscription, the fraction of CpG methylated promoters increased, we wondered whether this phenomenon is only a consequence of instability and loss of active rDNA repeats. To test this possibility, we performed quantitative PCR (qPCR) experiments monitoring relative rDNA abundance in wt, pre/B220^high^, pre/B220^low^, and tumor B cells. The two pre-malignant states harbored the same rDNA amounts as wt B cells, whereas lymphoma cells lost ~20% of their rDNA copies (Figure S2). Therefore, we concluded that the reduction in number of repeats, even if it would be selective for the transcription-permissive ones, could only partly account for the gain in promoter methylation. With regard to an active methylation mechanism, we followed up previous work showing *Myc*-dependent up-regulation of DNMTs in lymphoma [39]. Expression analysis of DNMT1, 3a and 3b revealed similarly low levels in wt and pre/B220^high^ B cells, whereas promotion (pre/B220^low^) and progression stages of malignant transformation were associated with a gradual elevation of all three DNMT mRNAs (Figure 2c). Based on this observation, we next asked how the surplus of DNMTs is harnessed for rDNA promoter methylation. Given that pRNA is the key player in targeting NoRC and associated chromatin modifiers to rDNA [19,20], we measured pRNA levels and found the same pattern as for the expression of DNMTs, i.e., pRNA levels were not altered in pre/B220^high^ B cells, moderately elevated in pre/B220^low^ cells and became strongly increased upon tumor progression (Figure 2d). Likewise, Pol I (RPA116) occupancy in the pRNA-encoding region upstream of the pre-rRNA start site was markedly enhanced in primary lymphoma and 152M cells (Figure 2e). The results show that upon lymphoma promotion, progression and maintenance rDNA silencing is reinforced by concerted up-regulation of DNMTs and pRNA.

### 2.3. Promoter Methylation Protects rDNA Stability in Lymphoma Cells

**Figure 3:**
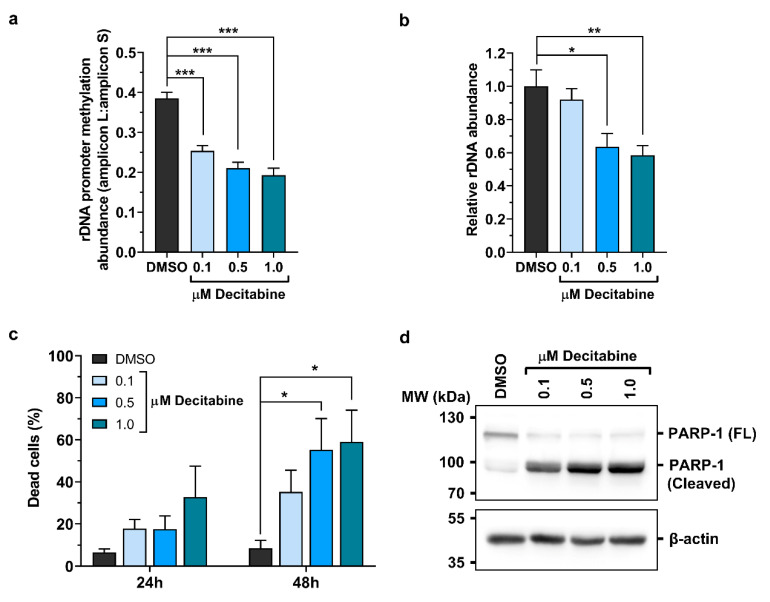
Death of Eµ-*Myc* lymphoma cells by promoter hypomethylation and destabilization of rDNA. 152M cells were treated with vehicle (DMSO) or with 0.1, 0.5, and 1.0 µM of the DNMT inhibitor Decitabine for 48 h. (**a**) Changes in rDNA promoter methylation between treated and untreated cells were monitored by DNA digestion with *Hpa*II and subsequent qPCR analysis of amplicons L and S. (**b**) Genomic DNA was used as template in qPCR with primer pairs for either the rDNA promoter or the *Rab5c* gene promoter. Abundance of rDNA was expressed relative to *Rab5c*. (**c**) Viability of cells treated with DMSO or Decitabine for the indicated times was monitored by Trypan Blue staining. The percentage of dead, membrane-compromised cells that were stained is displayed. For statistical analysis a two-way ANOVA test with Tukey multiple comparisons was used. (**d**) Immunoblotting of PARP-1 showing cleavage of the full-length (FL) protein upon Decitabine treatment. β-actin served as a loading control. Densitometric analysis and uncropped western blot images are provided in Figure S4a. Bar diagrams represent mean values ± SEM from at least three biological replicates. * *p* < 0.05, ** *p* < 0.01, and *** *p* < 0.001.

A recent study has shown extensive genome alterations upon Eµ-*Myc* lymphoma formation [40]. While genome instability drives cancer evolution, it also creates vulnerability to unsustainable lesions. Based on the high susceptibility of rDNA to recombination and rearrangements [24], we wondered whether augmented epigenetic silencing is required to safeguard genomic stability of rRNA genes. To test this hypothesis, we treated 152M cells with the general DNMT inhibitor Decitabine (5-Aza-2′-deoxycytidine), which is a Food and Drug Administration (FDA)-approved drug for therapy of myelodysplastic syndromes (MDS) [41]. Low amounts of Decitabine (0.1, 0.5 and 1.0 µM for 48 h) caused dose-dependent hypomethylation of the rDNA promoter as measured by *Hpa*II digestion, CpG methylation declining by ~50% with the highest dosage (Figure 3a). In accord with the loss of promoter methylation, the abundance of rDNA was also severely reduced. Cells treated with 1.0 µM Decitabine had ~40% less rDNA compared to untreated cells (Figure 3b). Decitabine treatment did not significantly affect pre-rRNA levels, suggesting that loss of epigenetic silencing and attrition of rDNA repeats counterbalance their opposing effects on the transcriptional output (Figure S3a). Importantly, CpG hypomethylation and genomic instability strongly impaired cell viability as assessed by Trypan Blue staining and cleavage of PARP-1, a bona fide substrate of several cell death proteases [42] (Figure 3c,d and Figure S4a). To further characterize the underlying death pathway, we monitored DNA damage-induced up-regulation of p53 [43,44]. However, p53 was not detectable in untreated or treated 152M cells, indicating that the p53-encoding *Trp53* gene was mutated (Figures S3b and S4b). Consistent with this notion and the frequently observed *Trp53* mutations in Eµ-*Myc* lymphomas [40,45], 152M cells harbored marginal p53 mRNA levels, further suggesting mutation-dependent down-regulation of *Trp53* (Figure S3c).

Taken together, these data strongly suggest that the epigenetically silent promoter configuration is essential to protect the integrity of rDNA repeats. Moreover, the concurrent up-regulation of Myc, DNMTs and pRNA during lymphomagenesis indicates that Myc may compensate transcriptional activation of one rDNA fraction by reinforcing silencing of the other fraction, thus ensuring overall rDNA maintenance. If this ‘rDNA equilibrium’ is perturbed, Eµ-*Myc* lymphoma cells are highly prone to cell death, even in the absence of functional p53.

## 3. Discussion

Myc is a key driver of ribosome biogenesis and protein synthesis, and thereby fuels sustained proliferative signaling and hypertrophy of nucleoli, i.e., two hallmarks of cancer [15,46]. The intimate link between Myc, ribosome biogenesis, and malignant transformation was imposingly demonstrated in the Eµ-*Myc* lymphoma model. Barna and colleagues showed that oncogenic properties of the Eµ-*Myc* transgene are suppressed in mice protected from aberrant protein synthesis due to ribosomal protein haploinsufficiency [16]. While this study pointed to a role of Myc in cancer initiation, Bywater et al. found that genetic or pharmacological interference with elevated rDNA transcription severely impaired proliferation and survival of Eµ-*Myc* lymphomas [47]. Therefore, Myc’s broad impact on the translation machinery seems to be crucial throughout carcinogenesis, however, the requirements may differ in the course of cancer development. In line with this notion, we observed striking changes in pre-rRNA synthesis during lymphomagenesis. The moderate increase in the initiation and promotion phases was followed by a drastic enhancement of pre-rRNA levels at the lymphoma stage. The difference between pre-tumoral and tumoral B cells in pre-rRNA levels followed the pattern of *Myc* expression and was in accord with Myc-dependent stimulation of Pol I transcription [11,12].

In spite of elevated pre-rRNA levels, Eµ-*Myc* B cells displayed increased CpG methylation at rDNA promoters, which is a well-established feature of the transcription-refractory state [22]. This finding indicates that, while the activity of permissive rRNA gene copies is boosted, the relative proportion of epigenetically silent repeats is enlarged. Similarly, it has been shown that Myc-driven rRNA synthesis in prostate cancer does not cause hypomethylation of rDNA promoters [35]. Given that expression of DNMTs and pRNA in pre/B220^high^ cells was comparable to wt B cells, the observed increase in CpG methylation remains obscure. An explanation may be provided by the direct interaction between Myc and the de novo DNMTs 3a and 3b [48,49], raising the possibility that these enzymes are co-recruited to rDNA by elevated Myc occupancy. With DNMTs and pRNA being up-regulated in pre/B220^low^ and lymphoma cells, the conventional, NoRC-mediated mechanism of rDNA silencing is likely to be reinforced upon tumor promotion. In this regard, the strong activation of the rDNA upstream promoter directing pRNA synthesis is an intriguing finding of our study. In non-transformed cells, the upstream promoter is only weakly transcribed by Pol I and does not respond to mitogenic stimulation [20,50]. We can envision two mechanisms of how excessive Myc levels facilitate pRNA production: On the one hand, oncogenic Myc levels occupy sites in the genome not bound by physiological Myc levels [10,33,51]. Accordingly, elevation of Myc abundance in pre/B220^low^ and tumor cells may lead to ‘invasion’ of novel rDNA sites and in turn upstream promoter activation. On the other hand, general rDNA chromatin opening in Eµ-*Myc* lymphomas has been shown [52], indicating an epigenetic ripple effect from the main to the upstream promoter. Both mechanisms are not mutually exclusive and may even act in concert, leading to the high levels of pRNA that we have observed in primary and cultured lymphoma cells.

Their high transcriptional activity, repetitive nature, and distribution over different chromosomes make rRNA genes especially prone to recombination and rearrangements. Epigenetically silent rDNA repeats are believed to function as a ‘buffer’ protecting genomic stability [19,20,23,24]. Accordingly, hypertranscription of rRNA genes in various cancers is associated with structural alterations and loss of rDNA repeats [53,54]. Likewise, we detected a reduction of rDNA content by ~20% in Eµ-*Myc* lymphoma cells. However, treatment with the DNA hypomethylating drug Decitabine strongly diminished rDNA abundance, corroborating that promoter methylation is indeed vital for rDNA integrity. Similar to our findings, a previous study reported rDNA destabilization upon genetic ablation of DNMT1 and DNMT3b in human colon cancer cells [55]. Moreover, Decitabine treatment of Burkitt lymphoma cells induces DNA damage, which is strongly dependent on *Myc* overexpression and leads to cell death even in a p53-deficient background [56].

Taken together, a picture of the Janus-faced role of Myc at rRNA genes is emerging. On the one hand, Myc induces hypertranscription and thereby jeopardizes genomic integrity. On the other hand, Myc directly and indirectly facilitates promoter hypermethylation, thus protecting rDNA stability. Interference with either of the two dichotomous functions, e.g., by inhibiting Pol I [47] or DNMTs, may be a suitable strategy for treating B cell lymphomas. While the Pol I inhibitor CX-5461 has currently completed Phase I clinical trials in patients with advanced hematological malignancies [57], specific hypomethylation and destabilization of rDNA has so far not been explored for cancer treatment. Future studies, for instance, employing inhibitors of the NoRC-subunit TIP5 [58,59] or therapeutic antisense oligonucleotides (ASOs) against pRNA, will advance tackling of this weak spot in Myc-driven cancers.

## 4. Materials and Methods

### 4.1. Eμ-Myc Mice and Isolation of B Cells

Wild-type C57BL/6J mice and genetically engineered Tg(IghMyc)22Bri (“Eµ-*Myc*”; MGI ID: 2447604) mice with C57BL/6J background [29] were housed in individually ventilated cages under specific-pathogen-free conditions. Mouse breeding (registration number 02-053/16) and experimental procedures were approved by the appropriate institutional and governmental committees for animal welfare (Thüringer Landesamt für Verbraucherschutz). All legal specifications regarding European guideline 2010/63/EU were followed. Female and male mice were sacrificed at the age of 8–10 weeks (wild-type and pre-malignant Eμ-*Myc* mice) or at 10–12 weeks when lymphomas were palpable (Eμ-*Myc* mice). B cells were isolated from spleen of wild-type and pre-malignant Eμ-*Myc* mice, while the tumor B cells were isolated from lymph nodes.

### 4.2. Fluorescence-Activated Cell Sorting (FACS)

Freshly isolated spleen or lymph nodes were crushed in phosphate buffered saline (PBS, 137 mM NaCl, 2.7 mM KCl, 10 mM Na_2_PO_4_, 1.8 mM KH_2_PO_4_, pH 7.4) between glass slides and the cell suspension was centrifuged at 700× *g* for 5 min. Sedimented cells were incubated in hypotonic red blood cell lysis buffer (155 mM NH_4_Cl, 12 mM NaHCO_3_, 0.1 mM EDTA) for 10 min at room temperature. Lysis was stopped by adding PBS and leukocytes were stained with fluorophore-coupled antibodies anti-CD19-FITC (Biolegend, San Diego, CA, USA), anti-B220-APC and anti-IgM-PE-Cy7 (both from eBioscience, San Diego, CA, USA) for 15 min at 4 °C and washed twice with PBS before sorting. Wild-type B cells were gated as single live CD19+/B220+ cells. The pre-tumor B cells were pre-gated on CD19+ and separated into two populations based on surface levels of B220 referred to as pre/B220^high^ and pre/B220^low^. Aliquots of tumor cell preparations from lymph nodes were stained for CD19, B220, and IgM, and only preparations containing at least 95% CD19+/B220^low^/IgM− cells were used. All flow cytometric measurements were performed using an LSR Fortessa system (BD Biosciences, San Jose, CA, USA) and data were acquired with BD FACSDIVA V8.0.1 (BD Biosciences, San Jose, CA, USA). B cells were sorted according to B220 expression using a FACS Aria III system (BD Biosciences, San Jose, CA, USA). FACS data were analyzed using Flowlogic (Miltenyi Biotec, Bergisch Gladbach, Germany).

### 4.3. Cell Culture and Decitabine Treatment

The cell culture adapted Eμ-*Myc* lymphoma cell clone 152M was maintained in RPMI 1640 medium (Sigma-Aldrich, Taufkirchen, Germany) supplemented with 10% (*v*/*v*) fetal calf serum (FCS), 50 µM β-mercaptoethanol (Carl Roth, Darmstadt, Germany), 10 mM HEPES and 100 units/mL penicillin and 0.1 mg/mL streptomycin (both Sigma). Cells were cultured under humidified atmosphere at 5% CO_2_ and 37 °C. For inhibition of DNA methyltransferases, cells were treated with 0.1, 0.5, and 1.0 µM Decitabine (MedChemExpress, Monomouth Junction, NJ, USA) for 48 h. Cell viability was monitored by Trypan Blue (Sigma-Aldrich, Taufkirchen, Germany) staining.

### 4.4. Gene Expression Analysis

Cellular RNA was isolated with peqGOLD TriFast Reagent (VWR Peqlab, Darmstadt, Germany) and residual genomic DNA (gDNA) was removed by DNase I (Sigma-Aldrich, Taufkirchen, Germany) digestion. cDNA was synthesized using random hexamers and SuperScript IV Reverse Transcriptase (Thermo Fisher Scientific, Waltham, MA, USA). Reactions without Reverse Transcriptase served as controls to exclude contamination with gDNA. Gene expression was measured by amplifying cDNA with transcript-specific primer pairs on a QuantStudio 3 qPCR instrument (Applied Biosystems, Foster City, CA, USA) using the Maxima SYBR Green/ROX qPCR Master Mix (Thermo Fisher Scientific, Waltham, MA, USA). Gene expression levels were normalized to beta-2-microglobulin (B2M) cDNA abundance. Primers for qPCR analysis are listed in Table S1.

### 4.5. Analyses of rDNA Methylation and Abundance

Cells were lysed and gDNA was isolated using Quick-DNA miniprep kit (Zymo Research, Freiburg, Germany) according to the manufacturer’s instructions. To monitor the methylation status of the cytosine at position −143 of the rDNA promoter, 100 ng gDNA was digested with the CpG methylation-sensitive restriction enzyme *Hpa*II (New England Biolabs, Frankfurt am Main, Germany) at 37 °C for 1 h followed with enzyme inactivation at 80 °C for 15 min. Afterwards, undigested, i.e., methylated, rDNA was qPCR-amplified with primers spanning the region from position −205 to −1. Amplification of an rDNA promoter fragment from −127 to −1 was used for normalization.

For measuring 5-hydroxymethylcytosine (5hmC) levels at position −143, 5hmCs in gDNA were glucosylated to confer resistance to the *Hpa*II isoschizomer *Msp*I using the Quest 5hmC detection kit (Zymo Research, Freiburg, Germany). After incubation with *Msp*I and DNA clean-up, the same qPCR analysis as described above was conducted.

The abundance of rDNA in B cells was quantified as described previously [60] with some modifications. An rDNA fragment form −205 to −36 was qPCR-amplified form 10 ng gDNA as template. The data were normalized to a unique gene desert region on chromosome 15. In the case of Decitabine treatment, the *Rab5c* promoter region was used for normalization due to Decitabine-induced genomic instability of the gene desert. All primers utilized in the gDNA analyses are listed in Table S1.

### 4.6. Chromatin Immunoprecipitation (ChIP)

ChIP assays in primary and cultured B cells were performed as described previously [61]. Briefly, cells were fixed in 1% formaldehyde for 10 min and quenched in 125 mM glycine for 5 min. Cell nuclei were isolated and disrupted in buffer containing 1% SDS. Chromatin was sheared in a Bioruptor Pico (Diagenode, Seraing, Belgium) to an average length of 200–500 bp. Chromatin was diluted, pre-cleared and incubated overnight at 4 °C with antserum against the second-largest Pol I subunit RPA116 [62]. Immune complexes were captured on Protein A/G-Sepharose beads (GE Healthcare, Chicago, IL, USA), extensively washed and eluted in elution buffer (0.1 M NaHCO_3_, 1% SDS). After decrosslinking at 65 °C for 6 h, DNA was purified with the ChIP DNA Clean and Concentrator Kit (Zymo Research, Freiburg, Germany). Immunoprecipitated and input DNA was analyzed by qPCR using primers pairs for different rDNA regions (see Table S1).

### 4.7. Western Blotting

Cells resuspended in cell lysis buffer (50 mM Tris-HCl [pH 8.0], 150 mM NaCl, 1% Triton X-100, 10 mM EDTA, 0.1% SDS) were lysed by sonication. Protein lysates were cleared by centrifugation and 40 µg of total protein were separated on a 10% SDS-polyacrylamide gel. After semi-dry blotting to a nitrocellulose membrane (Carl Roth, Darmstadt, Germany), immunodetection was carried out with primary antibodies against PARP-1 (Cell Signaling Technology, Danvers, MA, USA; #9542), p53 (abcam, Cambridge, UK; ab26) and β-actin (Sigma-Aldrich, Taufkirchen, Germany; A2066), followed by incubation with respective secondary antibodies coupled to horseradish peroxidase (HRP). The SuperSignal^TM^ West Pico Plus (Thermo Fisher Scientific, Waltham, MA, USA) chemiluminescent substrate was added and signals were recorded with a Fusion Solo S imaging system (Vilber Lourmat, Eberhardzell, Germany).

### 4.8. Statistics

For statistical testing unpaired two-tailed Student’s *t*-test with Welch’s correction was used unless stated otherwise. Analyses were carried out with Graphpad Prism software (Version 8.4.3). Data are presented as mean ± standard error of mean (SEM) from three or more independent biological replicates (* *p* < 0.05, ** *p* < 0.01, *** *p* < 0.001, and **** *p* < 0.0001).

## 5. Conclusions

Upregulation of *Myc* expression and of ribosome biogenesis are hallmarks of cancer. Both processes are intimately linked, as Myc is a major driver of ribosome production and also stimulates rRNA synthesis. Our study of B cell lymphomagenesis in Eµ-*Myc* mice has confirmed the oncogenic relationship between Myc and rRNA genes. However, we found that supraphysiological Myc levels in lymphoma cells also induce rRNA gene silencing, which in turn keeps instability and loss of these genes in check. Importantly, pharmacological impairment of rRNA gene silencing efficiently kills lymphoma cells, even in the absence of functional p53. Thus, we conclude that interference with heterochromatin formation at rRNA genes may be a promising strategy to treat B cell lymphomas as well as Myc-driven cancers in general.

## Supplementary Materials

Supplementary materials can be found at https://www.mdpi.com/2072-6694/12/10/3009/s1, Figure S1: 5-hydroxymethylcytosine (5hmC) levels at the rDNA promoter, Figure S2: Loss of rDNA in the course of lymphomagenesis, Figure S3: Impact of Decitabine treatment on pre-rRNA and p53 expression in 152M cells, Figure S4: Full-length scans of western blot images and signal quantification, Table S1. List of qPCR primers used in this study.
