# Supplementary material for: Dichotomous Impact of Myc on rRNA Gene Activation and Silencing in B Cell Lymphomagenesis
Source: Cancers (Basel). 2020 Oct 16;12(10):3009. doi: 10.3390/cancers12103009 (PMC7656300; doi:10.3390/cancers12103009)
Supplement: Supplementary file 1 [file cancers-12-03009-s001.pdf]

Supplementary Material

Dichotomous impact of Myc on rRNA gene activation and silencing in B cell lymphomagenesis

Gaurav Joshi, Alexander Otto Eberhardt, Lisa Lange, René Winkler, Steve Hoffmann, Christian Kosan and Holger Bierhoff

Supplementary Figures S1-S4

Supplementary Table S1

Supplementary Figure S1

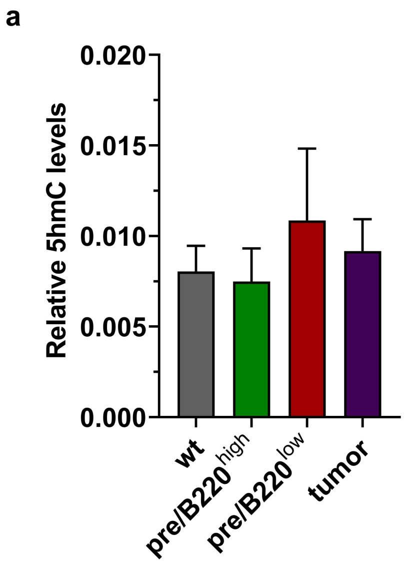

Figure S1. 5-hydroxymethylcytosine (5hmC) levels at the rDNA promoter. (a) 5hmC residues in genomic DNA from wt mice or pre-tumoral and tumoral Eμ-*Myc* mice were enzymatically glucosylated. The glucosylation conferred resistance to *Msp*I, which recognizes the sequence CCGG at positions -144 to -141 of the rDNA promoter. After restriction digestion, undigested (i.e. hydroxymethylated) rDNA was qPCR-amplified with primers -205/-185 and -21/-1 and levels were normalized to a promoter fragment (-127 to -1) not encompassing the restriction site.

Supplementary Figure S2

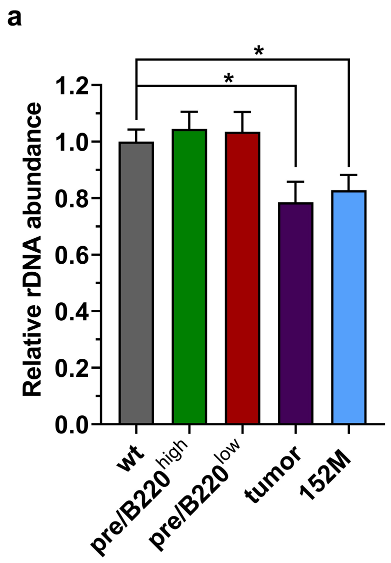

Figure S2. Loss of rDNA in the course of lymphomagenesis. (a) Genomic DNA from the indicated B cell populations was isolated and used in qPCR with primer pairs for either the rDNA promoter or a gene desert region on chromosome 15. Abundance of rDNA was expressed relative to gene desert. Bars represent mean values ± SEM from at least three biological replicates. *$p < 0.05$.

Supplementary Figure S3

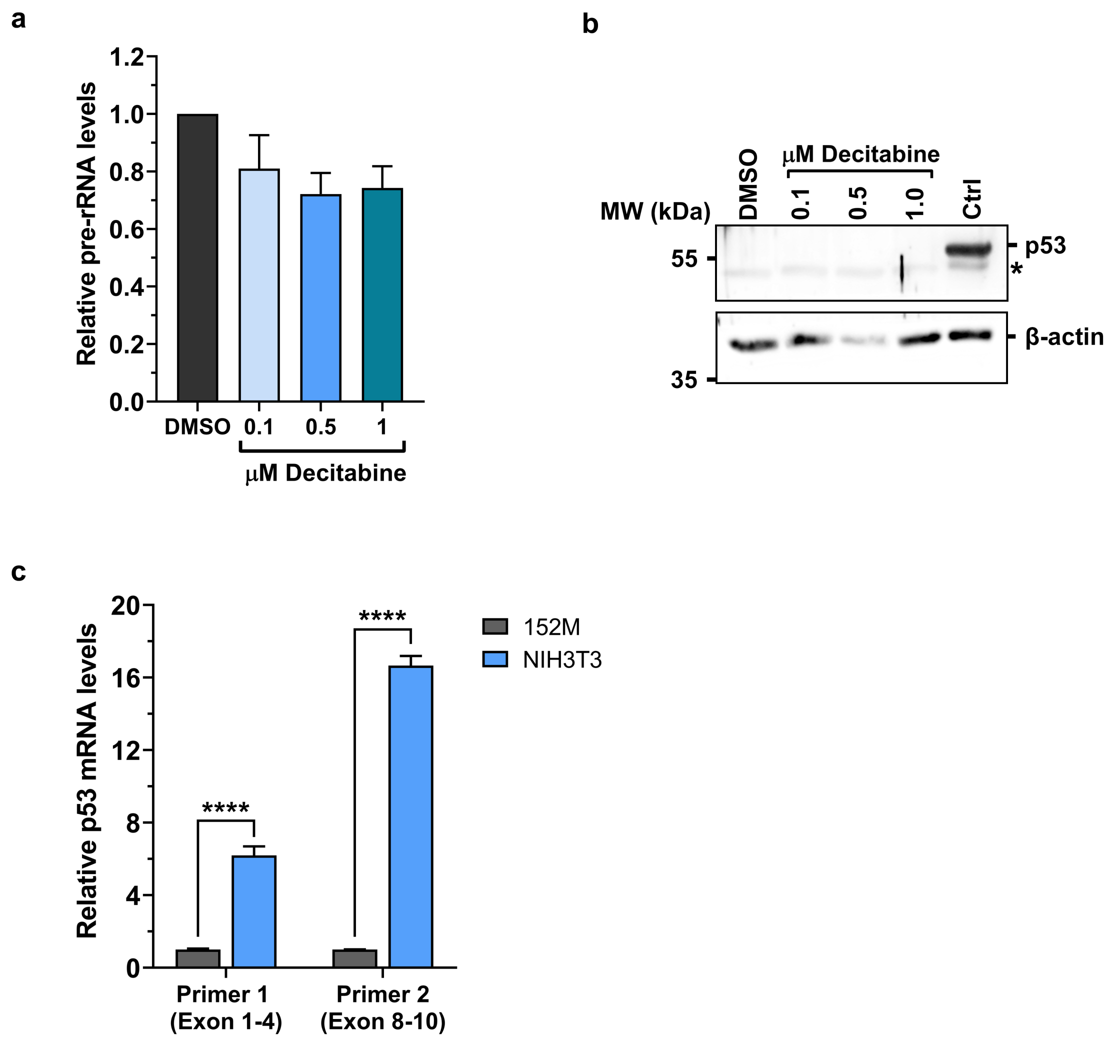

Figure S3. Impact of Decitabine treatment on pre-rRNA and p53 expression in 152M cells. (a) Cells were treated with vehicle (DMSO) or with 0.1, 0.5 and 1.0 µM Decitabine for 48 h. Abundance of pre-rRNA was measured after reverse transcription by qPCR and normalized to B2M mRNA levels. (b) Western blot analysis of p53 upon DMSO or Decitabine treatment. In the rightmost lane, a lysate from the glioma cell line U87MG treated with mycophenolic acid was loaded as a control (ctrl) for p53 detection. Immunostaining of β-actin was used as a loading control. The asterisk marks an unspecific band. Densitometric analysis and uncropped western blot images are provided in Figure S4b. (c) Comparison of p53 mRNA levels between 152M cells and NIH3T3 fibroblasts. After reverse transcription, the cDNA was amplified in qPCR with a primer pair spanning Exons 1-4 (Primer 1) or Exons 8-10 (Primer 2) of the p53 mRNA. Values were normalized to B2M mRNA levels. Bars represent mean values ± SEM from at least three biological replicates. ****$p < 0.0001$.

Supplementary Figure S4

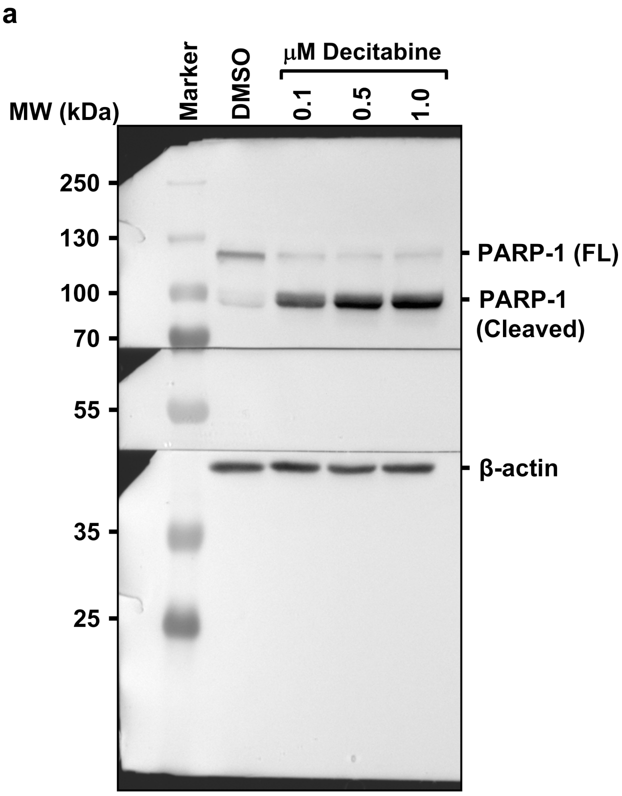

		Quantified signal (x10[6] a.u.)		
		PARP-1 (FL)	PARP-1 (Cleaved)	β-actin
Lane	DMSO	38.9	15.9	87.8
	0.1 µM Decitabine	12.2	98	86
	0.5 µM Decitabine	9.6	132.5	70.6
	1 µM Decitabine	12.8	145.9	79.9

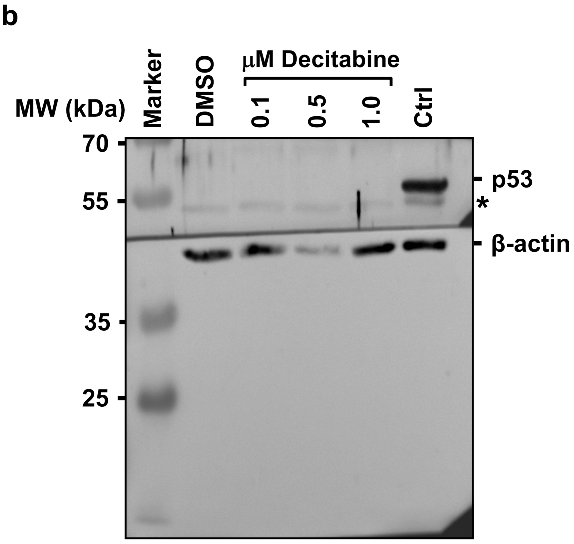

		Quantified signal (x10[6] a.u.)	
		p53	β-actin
Lane	DMSO	6.4	86
	0.1 µM Decitabine	1.8	71.4
	0.5 µM Decitabine	2.1	24.9
	1 µM Decitabine	6.8	80.3
	Ctrl	58.8	85.8

Figure S4. Full-length scans of western blot images and signal quantification. White light images of the marker bands and chemiluminescent images were recorded and digitally overlaid with the Fusion solo imaging software. Quantification of signal intensities was carried out with the same software and expressed as arbitrary units (a.u.). (a) Full-length scan of the immunoblot shown in Figure 3d. (b) Full-length scan of the immunoblot shown in Figure S3b.

Supplementary Table S1

Supplementary Table S1. List of qPCR-primers used in this study.

Region	**Sequence 5´ ---> 3´**
Primers for cDNA analysis	
*B220*_mRNA_Fwd	AGTGATGAACTGAGCACAACAGAG
*B220*_mRNA_Rev	GCAGCAGCGTGGATAACACA
*B2M*_mRNA_Fwd	CTGCTACGTAACACAGTTCCACCC
*B2M*_mRNA_Rev	CATGATGCTTGATCACATGTCTCG
*Dnmt1*_mRNA_Fwd	CAGTTGAAACTTCACCTAGTTCC
*Dnmt1*_mRNA_Rev	AGCACCACTCTCTGTGTCTAC
*Dnmt3a*_mRNA_Fwd	GAATTGTGTCTTGGTGGATGAC
*Dnmt3a*_mRNA_Rev	GTGGAATGCACTGCAGAAGG
*Dnmt3b*_mRNA_Fwd	ATGGAGATCAGGAGGGTATGGA
*Dnmt3b*_mRNA_Rev	GTCGCTTGGAGGTGGCTTTC
*Myc*_mRNA_Fwd	GTGCTGCATGAGGAGACACC
*Myc*_mRNA_Rev	AGGGGTTTGCCTCTTCTCC
pRNA_Fwd	GACCTGTCGGTCTTATCAGTTC
pRNA_Rev	ACCTATCTCCAGGTCCAATAG
pre-rRNA_Fwd	CGTGTAAGACATTCCTATCTCG
pre-rRNA_Rev	GCCCGCTGGCAGAACGAGAAG
p53 mRNA_Exon1-4_Fwd	TCTCCGAAGACTGGATGACT
p53 mRNA_ Exon1-4_Rev	AACAGATCGTCCATGCAGTG
p53 mRNA_ Exon8-10_Fwd	CAGGGAGCGCAAAGAGAG
p53 mRNA_ Exon8-10_Rev	CTCCCGGAACATCTCGAAG
Primers for gDNA analysis	
mrDNA -205/-185 Fwd	GACCTGTCGGTCTTATCAGTTC
mrDNA -127/-105 Fwd	TGGGGTCATTTTTGGGCCACC
mrDNA -56/-36 Rev	GATCACAAGCATAAAAGAGAC
mrDNA -21/-1 Rev	ACCTATCTCCAGGTCCAATAG
*Rab5c*_promoter_Fwd	CTGAAAGGCGGAAAGTGGAGG
*Rab5c*_promoter_Rev	CAGCAGCGTTACTTTGGGC
Gene desert_Fwd	AGGGACCTGACTGGTGACTG
Gene desert_Rev	GTCCTGTCTGCATCCCATTT
Primers for ChIP analysis	
mrDNA_MEn_Fwd	CCTCCAGAAGCCCTCTCTTGT
mrDNA_MEn_Rev	CAGCTGGCCGAGCCACACCGG
mrDNA_M0_Fwd	GTTGTCAAGGGTCGACCAGTTG
mrDNA_M0_Rev	GTGTCCTTTAGTGTTAATAGG
mrDNA_M5_Fwd	GACGACCCATTCGAACGTCTG
mrDNA_M5_Rev	CCTCTCCGGAATCGAACCCTG
mrDNA_M13_Fwd	ACCTGGCGCTAAACCATTCGT
mrDNA_M13_Rev	GACAAACCCTTGTGTCGAGGG
mrDNA_M16_Fwd	AGCATGTAGCAGTTGTAGGA
mrDNA_M16_Rev	TGGAGAGATGGCTCATCGGTT
